# The wide-angle lens of implementation science to improve health outcomes in criminal legal settings

**DOI:** 10.1186/s40352-025-00323-x

**Published:** 2025-03-11

**Authors:** Faye S. Taxman, Steven Belenko

**Affiliations:** 1 George Mason University, Fairfax, USA; 2 Temple University, Philadelphia, USA

**Keywords:** Implementation science, Inner and outer setting, Facilitators and barriers of change, Innovations

## Abstract

**Background:**

Implementation science (IS) is an emerging discipline that offers frameworks, theories, measures, and interventions to understand both the effective organizational change processes and the contextual factors that affect how well an innovation operates in real-world settings.

**Results:**

In this article, we present an overview of the basic concepts and methods of IS. We then present six studies where IS was used as a means to understand implementation of a new innovations designed to improve the health and well-being of individuals under criminal legal system supervision.

**Conclusion:**

We discuss how IS has developed new knowledge on how to implement efficacious innovations and suggesting future research needed to further our understanding of implementation and sustainability of innovations in the legal context.

The evidence-based practices and treatments (EBPTs) movement has led to greater clarity about what policies, practices, and treatments should be implemented to achieve desired positive outcomes based on research evidence. EBPTs exist in medicine, social work, education, criminal legal, and many other fields. For example, cognitive behavioral therapies, contingency management, and standardized validated screening and assessment tools are known EBPTs that are commonly implemented in substance use treatment. In the field of corrections, the same three practices and treatments are recommended along with a greater emphasis on offering an environment that uses human service principles to encourage participation in such programs and services. Knowing which EBPT to implement is the first step. But the more critical step is *how* to implement the policies, practices, and treatment to achieve the same results as in the research laboratory—this is a challenging endeavor because of the unique laws, regulations, resources, staffing, culture, and climate issues that exist within organizations and systems that often define *what* gets implemented and *how* it works in practice. Implementation with fidelity to the tested innovation(s) will bring us closer to the outcomes that mirror empirical findings.

Implementation science (IS) emerged as a distinct and unique set of methodologies, perspectives, and processes to address the reality that most implemented practices and treatments do not emulate the science upon which they were built. Morris et al. (2011) found that it takes an average of 17 years for a practice to be practiced and that even with this long trajectory, the clinical practices only adopt an estimated 14% of the science. IS offers frameworks, theories, measures, and interventions to understand both the change processes that are effective and the contextual factors that affect how well an innovation operates in real-world settings. As such, IS is distinct from process and outcome studies, which tend to focus on the EBPT or innovation that is being implemented, whereas IS focuses on the organization/systems, including leaders, staff, external partners, and resources that are involved in implementing and sustaining an innovation. IS goes inside the black box of innovation to understand what are the driving, restraining, and stabilizing forces that impact how an environment adapts and adjusts to innovation. By contrast, process and outcome studies typically focus on the innovation itself while attending primarily to client-focused outcomes. IS helps to identify the steps and skills needed to assess barriers and facilitators, tailor implementation strategies, monitor and evaluate efforts, sustain and scale change. The research questions in IS studies are designed to understand the process of change in an organization/system and the impact of the change at all levels, from staff to clients to stakeholders to program or system outcomes. IS is more than measuring the adherence to the innovation’s core features (fidelity); it explores how the organization, staff, stakeholders, and clients adapt to the innovation. As noted by Van Denise et al. (2023), implementation studies have been used to examine various diseases that affect people involved in the criminal legal system, including infectious diseases, substance use, mental health, co-occurring SUD and mental health, and other conditions. In these studies, the emphasis is on examining factors that affect implementation and/or implementation outcomes. Outcomes tend to include acceptability, feasibility, and reach. These studies provide better insight into the criminal legal context that affects the delivery of services.

Over the past decade, IS has emerged to better understand the implementation process. Several national initiatives have helped to illustrate the value of IS in behavioral health and justice contexts. These include three National Institute on Drug Abuse efforts: Criminal Justice-Drug Abuse Treatment Studies 2 (CJDATS-2; see Belenko et al., 2013); Juvenile Justice—Translational Research on Interventions for Adolescents in the Legal System (JJ-TRIALS) (see Knight et al., 2016); Justice Community Opioid Innovation Network (JCOIN; see Ducharme et al., 2021); as well as a new initiative by the National Institute of Justice focusing on implementation research (LaVigne, 2024). This paper explores IS studies occurring in criminal legal and/or health settings to illustrate how IS can help illuminate the processes and challenges of effectively implementing EBPTs or any innovation in these contexts. We provide an overview of IS theories, methods, interventions, and measures, review six examples of implementation studies that shed light on the contextual factors of implementation, and then end with recommendations regarding future research needed to further integrate IS into intervention research to improve the fidelity of EBPTs.

## Implementation science: a science of organizational and system change

**Fig. 1:**
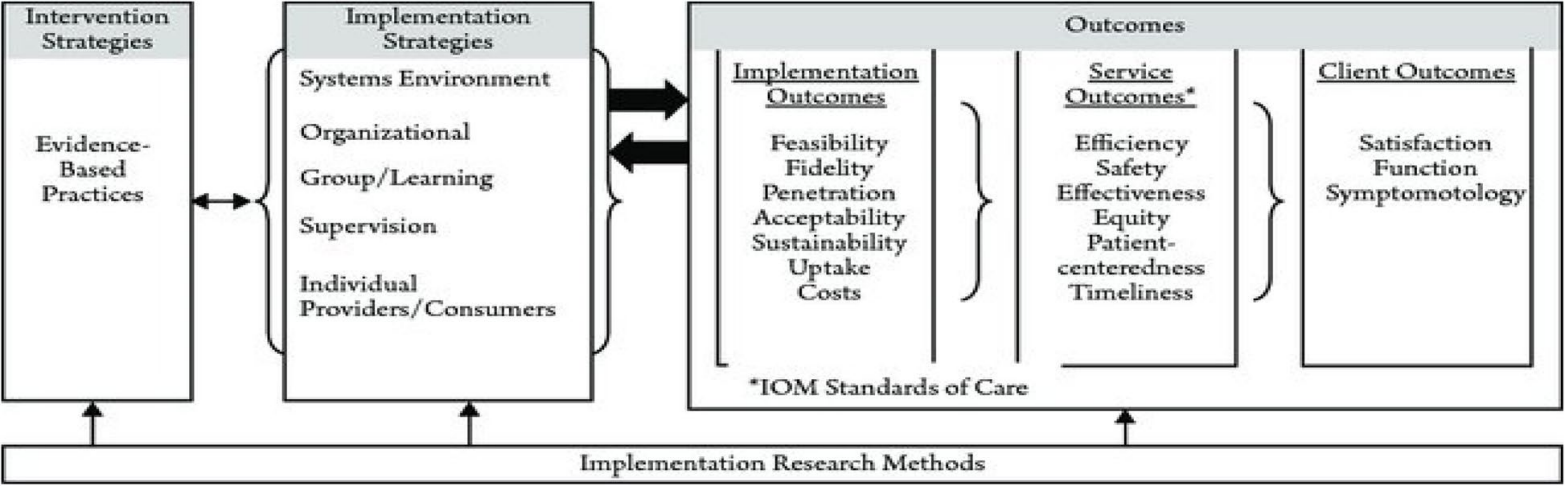
Proctor’s implementation outcomes framework (Proctor et al., 2009)

Fifteen years ago, Proctor et al. (2009) proposed that IS is distinct from other research methods, and provided a conceptual model to illustrate how IS can address innovation, implementation strategies, implementation outcomes, service outcomes, and various outcomes. As shown in Fig. 1, IS examines evidence-based policy, practice, treatment, or innovation with a specific purpose of understanding what is implemented, how it is being implemented, and providing tools to understand why and how an EBPT affects science as the scientific study of methods to promote the integration of research findings and evidence into healthcare policy and practice. IS posits that to most effectively implement an EBPT and understand how it operates in practice, we need to understand the organizational change processes. IS also provides specific interventions to guide organizations toward preparing for the adoption of an EBPT, implementing it, and sustaining it with fidelity over time. Powell et al. (2015) identified 78 different implementation strategies that have been used to implement changes in practices—while this is not the definitive list, it defines a starting place for considering what type(s) of organizational strategies are useful to address different innovation and contextual factors. Implementation strategies are techniques used to influence the organization and the staff and/or clients, particularly in terms of actions, attitudes, or behavior. The question of impact has to do with what type and how much impact has occurred due to the innovations being implemented. Proctor et al. (2009) define three types of outcomes: 1) *implementation*, which refers to a variety of organizational measures including staff and partner perception of the acceptability, appropriateness, and/or feasibility of the innovation; measures of adherence or fidelity of the innovation; degree to which the innovation is used including uptake, penetration, and/or sustainability; and costs; 2) *service-related* outcomes that have to do with organizational issues of efficacy, safety, procedural justice, and equity; and 3) *client-level* outcomes related to improvements in condition, short/long term results, and changes in functioning. Proctor et al. (2009) established that research can be conducted at each level of the model independently or with multi-level components that examine the impact of one component of the model on another component. A recent scoping review (Proctor et al., 2023) revealed that in the past decade after this model was specified, implementation studies have been conducted in a variety of settings with varied populations and various outcomes. While implementation outcomes are still infrequently measured, fidelity (similar to the concept in process evaluations) is the most likely to be measured (Proctor et al., 2023).

From a research methods perspective, Curran et al. (2012) identified how the flexibility in the Proctor model can lead to three types of implementation and effectiveness studies: (1) the Hybrid 1 type trial tests the effects of a clinical intervention on relevant outcomes while observing and gathering information on implementation; (2) the Hybrid 2 model tests both the innovation/clinical initiative and implementation interventions/strategies used in the implementation of the innovation on various outcomes; and (3) the Hybrid 3 model tests the implementation strategy while observing and gathering information on the clinical intervention’s impact on relevant implementation, service, and client-level outcomes. Each hybrid model offers opportunities to conduct rigorous research that can include using randomized controlled trials at the client/organizational level, clinical/innovation, implementation strategy, or multisite cluster randomized designs at the agency level. Each hybrid model offers potential methods for examining the implementation of an innovation and exploring various levels of outcomes. The flexibility is the unit of analysis– at the individual, provider/staff, or organizational level and whether the outcomes refer to staff attitudes to implementation outcomes (i.e., adoption, adaption, cost), service outcomes to health-related outcomes, or individual level outcomes.

## Frameworks for implementation

**Fig. 2:**
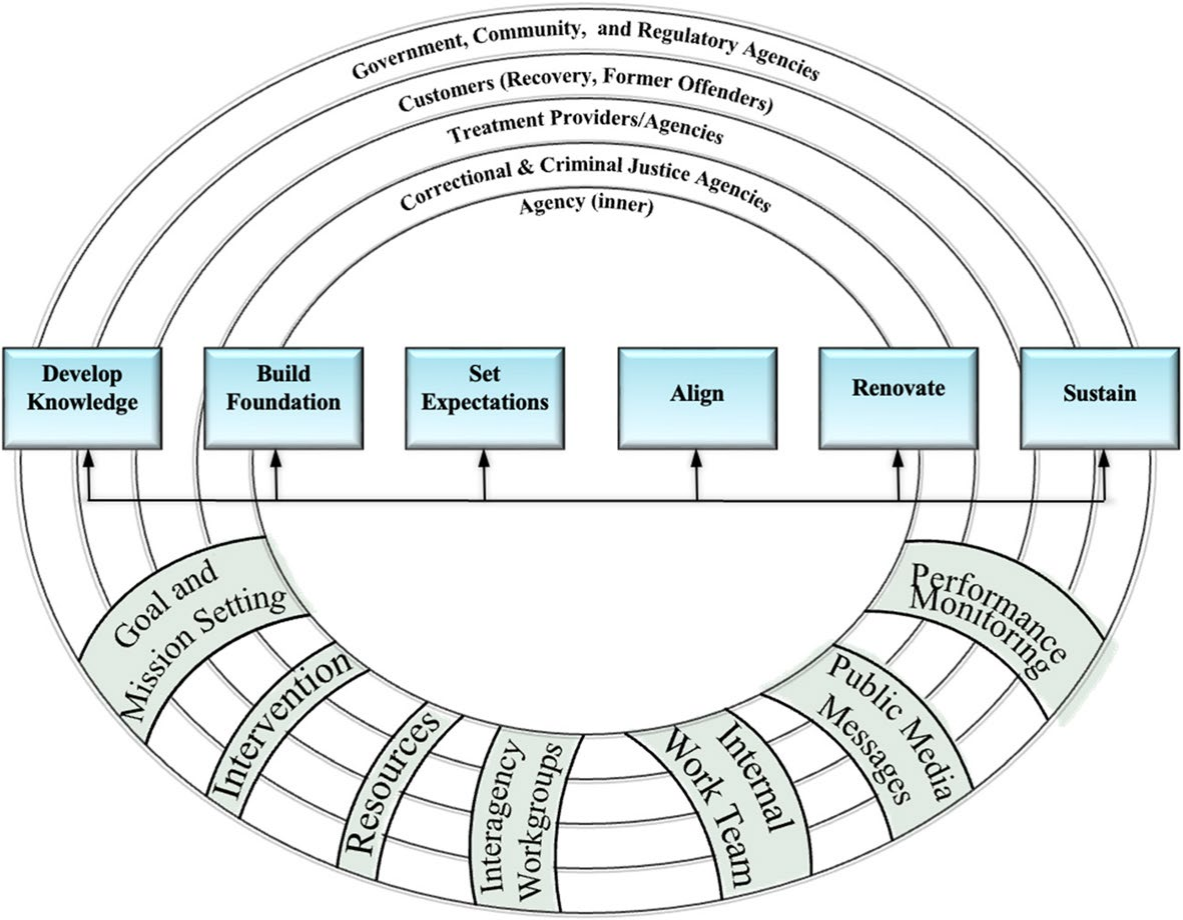
CJ IIM model

Besides recognizing the rich opportunities to examine which innovations fare better (in terms of client and organizational outcomes) in different contexts (e.g., settings, staffing, client characteristics, etc.), several frameworks have been developed to further an understanding of the implementation process and the factors that affect implementation and various types of outcomes. The change strategies that Powell et al. (2015) identified usually refer to a single implementation strategy. However, IS scientists recognize that implementation is a process often consisting of several steps or phases, with multi-level factors that influence implementation at each phase. This realization has helped to conceptualize implementation strategies as a sequence of events (often not linear) that begin with setting the stage for the innovation to focus on maintaining the innovation after it has been successfully implemented. Many models exist and they tend to have similar features that incorporate the domains articulated by the **Consolidated Framework for Implementation Research** (CFIR) (Damschroder et al., 2009): the innovation characteristics, inner setting, outer setting, process of change or implementation strategies, and individuals. Inner setting refers to the staff, leadership, culture, regulations, etc., and external/outer setting refers to supporting stakeholders that are vested in the outcomes—both the inner and outer settings consist of various contextual factors that can be viewed as similar to Lewin’s force field analyses and change model of driving forces, restraining forces, and equilibrium (Hussain et al., 2018). For example, Taxman and Belenko (2012) offered the **Criminal Justice Evidence-based Interagency Implementation Model** CJ(IIM) (Fig. 2), which addresses the unique features of legal settings regarding dissemination and implementation. The CJ-IIM recognizes that it is important to have inner (agency support) and outer (stakeholder) support to advance successful reform of EBPP in CJ. Buy-in from constituencies for CJ to pursue public health goals is critical. The CJ-IIM suggests that addressing contextual layers will facilitate EBPP adoption and implementation. CJ-IIM recognizes that there are various steps to implementation, including building knowledge, developing a foundation in the agency, setting expectations about the “value-added,” aligning with existing work processes, renovating work processes, and sustaining efforts to use the innovation as routine practices. The strategies identified in the CJ-IIM should meet the challenges of cross-contextual-layer reforms needed for behavioral health implementation in CJ systems which include change teams, resources, goal setting, performance-driven, and other implementation activities.

**Fig. 3:**
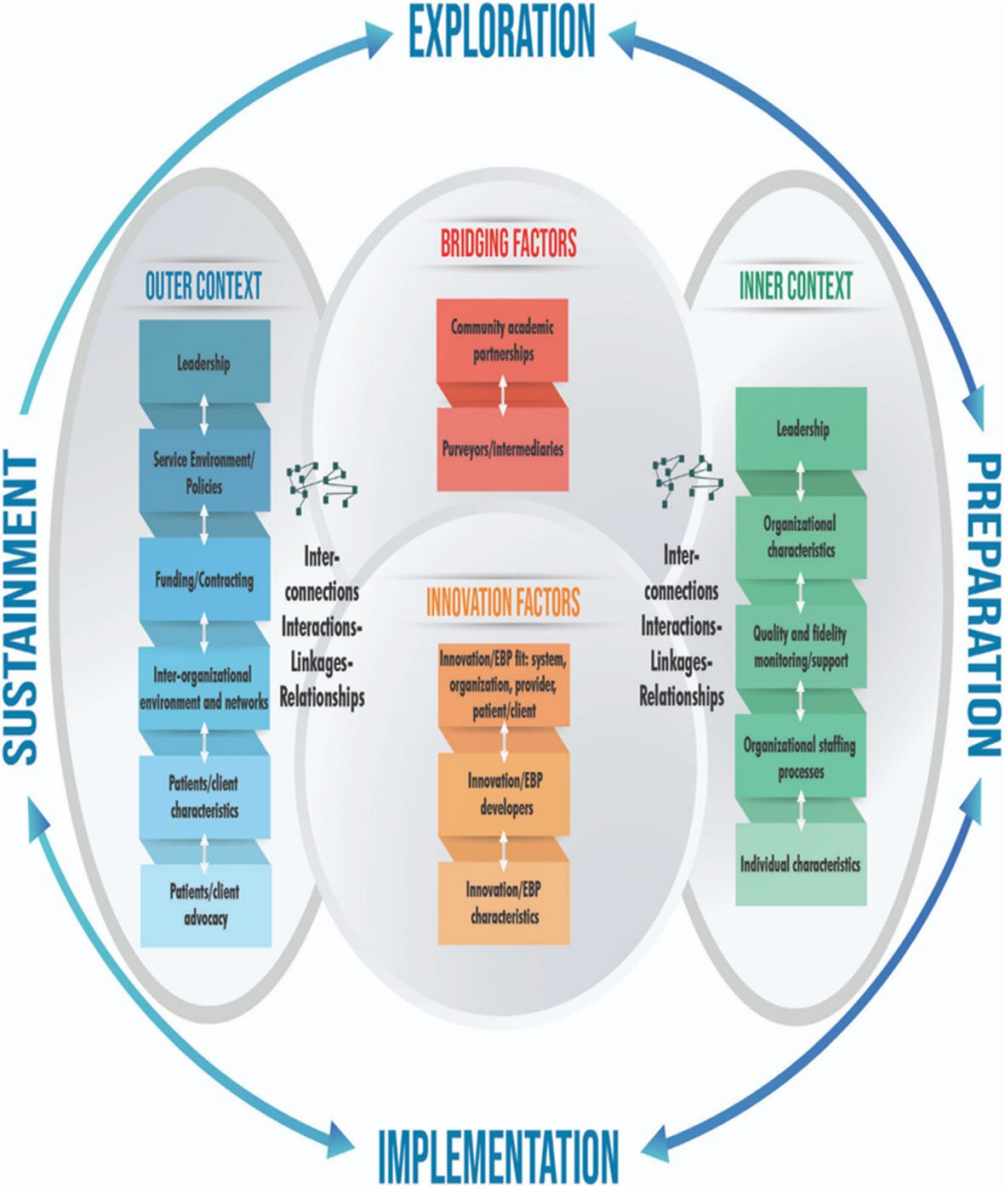
EPIS model

The CJ-IIM was developed through informant interviews, multi-layered organizational surveys (staff to administrators to external treatment providers), studies on behavioral health care in legal settings, and reviews of other IS frameworks such as the **Exploration Preparation Implementation, Sustainment** (EPIS) model (Aarons et al., 2011; see Fig. 3). The current iteration of the EPIS model now consists of four stages: exploration, preparation, implementation, and sustainment, as well as bridging factors (relationship among stakeholders) and innovation factors (features of the innovation) that affect how change can be pursued (Moullin et al., 2019). EPIS has been implemented in various child welfare settings, including one involving the juvenile legal system (see Knight et al., 2016), and health equity (Stanton et al., 2022). EPIS is one of the widely used frameworks, and a recent systematic review found 44 studies that were published using the EPIS framework where nearly 90% examined inner context factors, 57% examined outer context factors, 37% examined innovation factors, and 31% bridging factors (Moullin et al., 2019). The authors also report that most projects examined two phases of the EPIS model with nearly 75 percent measuring implementation.

## Examples of implementation studies in criminal legal setting contexts

Below we showcase several implementation studies using various types of designs to illustrate how IS can assist in furthering our understanding of how to implement innovations that are likely to have an impact on desired outcomes. The goal is to illustrate some of the implementation strategies used as well as the types of outcomes generated from the studies. The studies were selected based on their unique nature as well as their contributions to understanding different facets of the CFIR domains.

## Collaboration between drug courts and MOUD providers (adapted from Pivovarova et al., 2023)

Drug courts are designed to prioritize treatment and recovery as part of the case adjudication process. With judges overseeing the clinical care of individuals and working with a myriad of court actors (i.e., court coordinator, prosecutor, defense attorney, probation, etc.) and treatment providers, the drug court judge has a pivotal role in reviewing progress from treatment, modifying treatment plans, and using sanctions and rewards to incentivize recovery (Belenko, 2019). Drug courts are known to reduce recidivism, although there is a dearth of information about the impact on drug use (Faragó et al., 2023; Mitchell et al., 2012). Similar to other legal system efforts, drug courts tend to underutilize medications for opioid use disorders (Matusow et al., 2013). A recent study found that 86% of drug courts allowed participants to use medications but an average of only 14% of eligible drug court clients received medications (Farago’ et al., 2023); this is consistent with findings by Smith et al. (2024) that the average drug court used only 3 of the 10 key components of drug courts (Hiller et al., 2010; Office of Justice Programs, 2004).

The failure to adopt the ten standards of effective drug courts suggests widespread implementation issues in drug courts, as well as a need to understand the factors that affect the utilization of the standards, including medications. Pivovarova et al. (2023) conducted a quasi-experiment to explore the facilitators and barriers to the use of MOUD in seven drug courts in one northeastern state to understand the implementation factors and implementation strategies used by the drug courts. The study involved interviewing drug court staff using the **Consolidated Framework for Implementation Research** (CFIR) to identify the court-related operational issues, collaboration issues with treatment providers and other legal agencies, and beliefs and opinions of staff that might affect the use of medications. In other words, the study focused on understanding the inner and outer contexts that impact whether individuals in the drug court are referred for medications for their opioid use disorders. This topic is important because the state where the study took place has mandated that individuals incarcerated in jail be required to continue medications if they were on opioid use disorder medications in the community and/or provided with medications while incarcerated—a setting where one would presume the court and legal system would be amendable to the use of medications.

### Facilitators of MOUD interventions

The findings revealed several inner and outer setting issues that had a positive impact on the use of MOUD. Most important to the drug court officials was having pre-existing relationships with jails that provide MOUD, and service provider agencies that offer a range of behavioral health and social services including MOUD. Providers perceive that being responsive to the needs of the court and willing to work with the court on sensitive matters including the use of sanctions and modifying treatment based on the needs of court staff. These preferences reflect a desire to work with organizations that value the provision of higher quality services as well as using MOUD.

### Barriers to use of MOUD

Drug court professionals expressed several reservations about providers that affected their willingness to use MOUD, especially given the concerns about the prescribing practices of some providers and the perceived overmedication of clients. The drug court professionals revealed hesitation about the provider community overall, and how the providers work with individuals in the legal system. This is complicated by communication issues such as the hesitation of providers to share treatment progress information, including adherence to the medication schedule or attendance at behavioral therapy sessions. Some providers do not have information release procedures, lack personnel to handle progress reports, and do not prioritize the needs of the court—all contribute to concerns about the providers themselves and the quality of services provided.

A complicating factor is the role and use of behavioral therapies alongside medications. Behavioral therapies, particularly evidence-based cognitive behavioral therapy, are considered the primary treatment in drug court settings. Drug courts do not necessarily understand that, unlike buprenorphine and naltrexone, methadone is the only FDA-approved medication that requires counseling. Yet drug courts view behavioral therapies as critically important and feel that providers are negligent if behavioral therapies are not provided.

Another provider-related issue is the accessibility of providers to the clients. In a national survey of drug courts, it was noted that courts were more likely to have clients that use medications when a provider is local and accessible (Farago’ et al., 2023). The lack of availability of providers in the local community impacted the court’s willingness to recommend that clients take medications.

This study resolved several unanswered questions about the relationship between community treatment providers and drug courts that generally cannot be addressed in traditional process or outcome studies. The use of CFIR assisted in focusing attention on the relationship between the courts and providers, including an examination of factors within the drug courts and those that had to do with the bridging factors and stakeholder perspectives. It also allowed for an appreciation of how the attitudes and opinions of drug court personnel affect their willingness to use the MOUD innovation. The results also suggested that a key implementation strategy would be to focus on the collaborations and misconceptions, such as the use of behavioral therapies, the need for progress reports, and release procedures. Accordingly, the study uncovered issues that are not necessarily apparent by simply conducting clinical intervention studies that focus on client outcomes.

## Use of MOUD in jails (adapted from Molfenter et al., 2021, 2025)

With nearly 11 million people admitted each year, jails tend to have the largest concentration of opioid users (Matsumoto et al., 2022; Maruschak et al., 2023) than other criminal legal settings, thus providing an opportunity to provide treatment for opioid use disorders (OUD). But jails are generally chaotic environments where the average person spends 72 h in custody before being released to the community. Treatment services are relatively limited in jails given their low resources, high client turnover, and lack of medical and behavioral health staff. In states with the highest rates of opioid overdose deaths, 22% of individuals entering jail settings screened positive for OUD (Scott et al., 2021). FDA-approved OUD treatment medications–methadone, buprenorphine, and naltrexone–are considered the gold standard for treating OUD in general and with criminal legal populations. A large treatment gap exists, however, with just 9–15% of incarcerated individuals having access to these medications (Springer, 2024). Moreover, legal settings seldom provide aid to individuals with OUD to transition into the community and continue engagement in MOUD. The question is how to prepare jails to be a service provider.

Molfenter and colleagues (Molfenter et al., 2021) implemented a Hybrid 3 implementation effectiveness randomized controlled trial to test two implementation strategies, organizational coaching and Extension for Community Healthcare Outcomes (ECHO), offered at various dosage levels using the **Reach, Effectiveness, Adoption, Implementation, and Maintenance** (Re-AIM) framework. Both are considered successful evidence-based innovation (EBI) implementation for MOUD adoption. *NIATx Organizational Coaching* focused on action planning and goal setting as tools to address barriers and implement innovations. This consists of multiple activities such as training, support on change management, and how to apply the targeted innovation. *ECHO* is an implementation strategy that builds clinician capacity to adopt and perform the innovation. The model begins with a series of tele-video sessions that include a mix of didactic materials and case conferencing to address issues faced by clinical staff. In their comparative implementation effectiveness trial, Molfenter et al. (2021) examined the impact of MOUD use in jail and post-jail community-based treatment provider (CBTP) settings. The study consisted of 48 jails and CBTPs to determine the combination of coaching and ECHO implementation strategies. The implementation effectiveness trial had four study arms to compare Low-Dose and High-Dose NIATx Coaching, with and without ECHO, as shown in Table 1. The outcome variable was access to MOUD. The study hypothesized that sites assigned to the study arm involving high-dose NIATx coaching and ECHO will be the most successful in implementing or expanding MOUD use.

**Table 1:** Study arms in NIATx coach vs. Echo comparative implementation effectiveness trial

ARM	NIATx Coach	ECHO
High-Dose NIATx Coaching & ECHO	• Four-hour, Virtual Kick-Off Meeting split into two days with Study Team & Coaches • 12 monthly (one-hour) coaching calls with Change Leader/Team	• Prescribers participated in 12 monthly (one-hour) scheduled video conference calls
Low-Dose NIATx Coaching & ECHO	• Four-hour, Virtual Kick-Off Meeting split into two days with Study Team & Coaches • Four (one-hour) coaching calls at months 1, 4, 8, and 12 with Change Leader/Team	• Prescribers participated in 12 monthly (one-hour) scheduled video conference calls
High-Dose NIATx Coaching Only	• Four-hour, Virtual Kick-Off Meeting split into two days with Study Team & Coaches • 12 monthly (one-hour) coaching calls with Change Leader/Team	Not Offered
Low-Dose NIATx Coaching Only	• Four-hour, Virtual Kick-Off Meeting split into two days with Study Team & Coaches • Four (one-hour) coaching calls at months 1, 4, 8, and 12 with Change Leader/Team	Not Offered

### Change teams

The NIATx model requires each site to put together a change team, preferably an interagency team from jail and community agencies. In this study, each site had an Executive Sponsor, Change Leader, and Change Team. Members of the Change Team included criminal legal staff (jail or probation), health provider representatives, medical providers/prescribers (i.e., nurses, physicians), counselors, and other stakeholders to ensure that the team’s reach was extended to various pertinent audiences. The Change Teams work on quality process improvement projects using Plan-Do_Study-Act (PDSA) cycles to implement or improve MOUD practices and policies based on the study aim(s) the site identified at the start of the study.

A goal is a specific, measurable, achievable, relevant, and time-bound action, or SMART goal (Bailey, 2017). Some sites had one goal during the full study, whereas other sites identified two or three goals throughout (Table 2). The goals included (1) increasing the number of OUD screenings, (2) setting up an opioid treatment program (OTP) within the jail, (3) adding buprenorphine inductions to the existing MOUD program, (4) transitioning from Suboxone to Sublocade to improve efficiencies including staff time & diversion, and (5) increasing the number of individuals who connect with a community MOUD provider. Six coaches provided 10 h of virtual training and workgroup sessions.

**Table 2:** Site Goals for the NIATx vs. ECHO Study (Clark et al., 2023)

**Screened**
Improve/implement screening procedures to be able to identify individuals with OUD
Increase the number of screenings with incarcerated individuals to identify OUD (*n* = 5)
**In need of treatment**
Implement MOUD within the jail
Setup an opioid treatment program (OTP) within the jail
Increase the number of individuals connected to MOUD through use of flyer/hotline number
**Referred to treatment (none)**
**Initiation of treatment**
Increase the # of individuals receiving MOUD medication (*n* = 4)
Add buprenorphine induction to existing MOUD and scale it to all appropriate residents (*n* = 5)
Increase MOUD treatment with buprenorphine for those in need
Transition from suboxone to sublocade to improve efficiencies including staff time/diversion(*n* = 2)
Co-staff all incoming bookings with community service providers and jail medical to increase number of people on MOUD (*n* = 2)
Hire a medical doctor (MD) to begin doing MOUD initiation/inductions
Increase nursing staff to expand capacity for MOUD care
Make peer support more consistently available to those with OUD
**Treatment engagement (in corrections facility)**
Increase interdisciplinary jail staff communication and coordination of care for MOUD patients
Increase the number of individuals continuing with suboxone while incarcerated
Increase the number of inmates staying on buprenorphine when transferred to other DOC facilities rather than taper off
**Continuing care (transition to community)**
Improve the warm handoff to local community treatment providers upon release (*n* = 3)
Increase the number of community partners the jail has connections to
Increase connection to community resources upon discharge (*n* = 8)
Increase the rate in which individuals relate to community MOUD clinic (*n* = 3)
Create a bridge script protocol for release to the community (*n* = 2)
Implement care navigators to improve linkage to MOUD in the community
Increase the distribution of naloxone kits at release for individuals who request or have been diagnosed with OUD
Increase community providers presence in jail to improve coordination of care upon release
Increase jail staffs’ knowledge and awareness of MOUD services and providers in the community
**General**
Create formal protocol for pregnant women (*n* = 2)
Track those suspended from jail program for violations and to create consistent policy
Track recidivism back to jail of previous jail MOUD patients

Findings from this comparative implementation effectiveness trial revealed that low and high-dose coaching did not impact MOUD utilization (see Molfenter et al., 2025). A review of the ECHO intervention revealed that most of the issues that affect the uptake of medications in jails are related to administrative and jail culture rather than clinical issues. The ECHO-sponsored sessions, tailored to the needs of the audience, primarily focused on providing services in jail in terms of gathering officer support, delivery processes within a jail environment, and working with the jail administration; these are system issues compared to issues related to clinical care.

## Suicide prevention in an interagency collaboration (adapted from Elkington et al., 2023)

Youth involved in the juvenile legal system are at elevated risk for suicidal behaviors, yet few probation agencies screen for this risk. One of the barriers is that juvenile legal agencies do not have the capacity to respond to suicide risk behaviors, and mental health agencies do not prioritize these youth for services. To address these interagency issues—youth probation screening youth and triaging youth with mental health agencies on the need for services—Elkington et al. (2023) developed an interagency client decision-support system that bridges youth probation and mental health agencies. This system was supported by an app that automatically includes the triage formula to guide probation and mental health agencies on whether there is an urgent need for a mental health intake. The triage process is unique to each county given the site variation in services available in the county, as well as the capacity of the agencies to provide care. Each county could identify how to deliver priority services for those at great risk for suicide, including the means to transport the youth from the probation office to the treatment provider (i.e., ambulance, co-responder, police, etc.).

This implementation effectiveness study documents the pathway processes and fidelity to such processes on immediacy of care for high-risk youth, access to treatment services, and duration of treatment. The study focused on the pathways that were developed and fidelity to the triage process for ten jurisdictions in a northeastern state. The implementation intervention included an interagency workgroup consisting of administrators and staff from probation and behavioral health agencies. The workgroup defined the triage process, ensured that the transportation from the probation to the treatment agency used the most reliable method in the county, altered the screening process in the jail to use eConnect (a decision-support system that includes a screener for suicide risk behaviors), and altered the intake processes of the mental health agency. The study used the **Consolidated Framework for Implementation Research** to document the inner and outer setting issues. In addition, the study collected effectiveness data on youth treatment outcomes of initiation, engagement, and completion. Barriers to implementation were addressed during the workgroup processes.

**Fig. 4:**
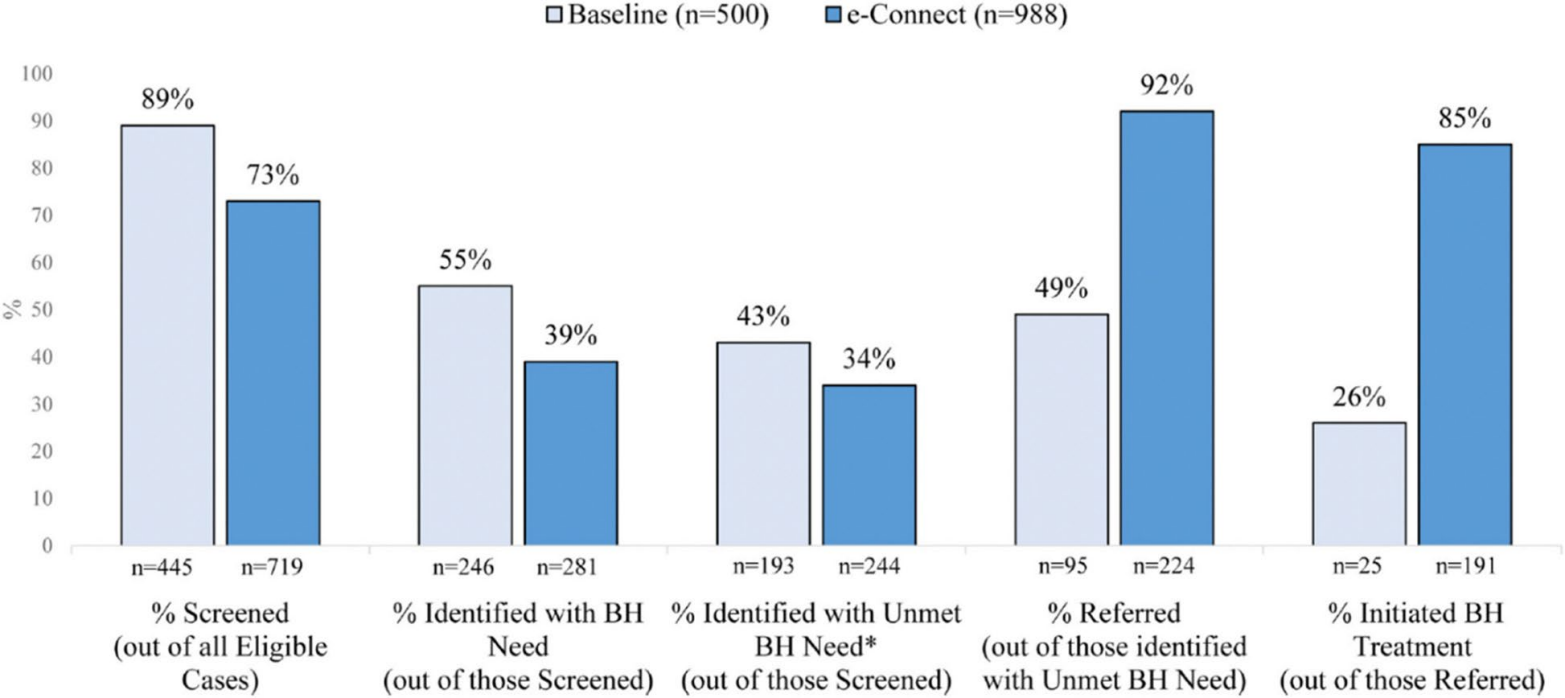
Results from eConnect on the Cascade of Care Ryan et al., 2023

The interagency workgroup intervention illustrates how the triage process can help advance the referral from probation to mental health treatment. In every part of the process, there were gains in the degree to which youth are managed by probation and/or treatment agencies. Ryan et al. (2023) reported a 92% referral rate, compared with 49% at baseline, and 85% initiated treatment compared with only 26% at baseline (Fig. 4). The development of the digital app to handle the triage process was viewed as successful. Even more importantly, while the baseline showed how Black boys and girls were significantly less likely to initiate treatment, the follow-up revealed that nearly all the youth appeared for treatment and found that most efforts resulted in the youth initiating treatment. Even more significantly, the process is race-neutral, with both parties likely to begin treatment.

## Mandated MOUD use in jail (adapted from Evans et al., 2021)

In 2018, the state of Massachusetts mandated that individuals with opioid use disorders in jail should be offered medications, and if they were on medications in the community, they should be allowed to continue the medications while incarcerated. The legislative mandate requires that all three FDA-approved MOUDS (i.e. naltrexone, buprenorphine, methadone) should be offered. A team from the University of Massachusetts Baystate and other institutions are working with seven counties, the MA Department of Public Health, and community treatment providers to implement a Type 1 effectiveness-implementation study regarding the legislative mandate (Evans et al., 2021). The study measures the outcomes of 7500 incarcerated individuals regarding the use of MOUD, retention in MOUD, and discharge status to track MOUD treatment initiation and engagement in jail and in the community following release, as well as recidivism. But more importantly, as a Type 1 hybrid trial, the emphasis is on understanding the implementation process in each jail in terms of providing medications as well as supporting the continuation of MOUD in the community. Implementation processes are assessed through surveys and qualitative interviews of staff and administrators in the jail and community provider programs using the **Consolidated Framework for Implementation Research** (CFIR). The qualitative interviews were designed to illuminate the inner and outer contexts that affected the use of MOUD in different jails and community treatment providers. Surveys collected information on the knowledge and attitudes towards MOUD by staff and administrators. The effectiveness of the implementation of the state mandate was assessed by tracking the outcomes of the 7500 individuals who were incarcerated and released during the study period.

Baseline surveys and interviews were used to understand the contextual factors around the perceptions about MOUD by staff/administrators and the inner and outer settings that might have an impact on MOUD use. First, 61 clinical staff, correctional officers, and jail administrators were interviewed about the legislative mandate regarding providing MOUD to those incarcerated in the local jail. These interviews showed that the legislative mandate improved the receptivity to MOUD being offered to people that are housed in the local jail (Pivovarova et al., 2022). The interviews revealed that the legislative mandate, along with funding for the medications, was persuasive for those who did not favor the availability of MOUD. Besides having an impact on the availability of MOUD in jails, the legislative mandate also affected the continuity of care regarding MOUD. However, it also presented certain challenges and struggles, primarily around the requirement to offer medications within 30 days of release for men. This was complex because of the uncertainty around release dates, particularly for individuals in pretrial status.

More importantly, the interviews revealed factors that had to be addressed in terms of administering MOUD in jail settings to comply with the mandate. Each medication presents challenges due to federal requirements regarding who can administer the medication as well as how it is administered (the requirement occurred before the recent change in regulations to allow for take-home doses of methadone from the previous regulations requiring individuals to receive daily dosages and monthly counseling by providers certified to dispense methadone). Buprenorphine requires a physician or nurse practitioner to administer the medication. Many jails decided to contract for the medication instead of having existing staff administer the medications. The contracting process contributed new administrative burdens including issuing a contract, having an officer to oversee the contract, and integrating the external staff with the jail staff, for example. The contractual staff had to get used to the routines of the jail, the security issues, and the chaotic nature of the jail environment. Other jails decided to transport people in jail to community providers to receive their medications, which required obtaining a van, staff, and funds for the transport. Some jails decided to offer methadone and outpatient therapies in-house; this required the jail to be certified as a provider, which requires procedures to disseminate medications and spaces for dosing (to observe taking methadone). Further inner setting issues had to do with the perspectives and opinions of correctional officers and medical staff—overall, there was a need to understand that both officers and medical staff had little experience with MOUD, and therefore, there was a need to train both staff on the medications and their value. Another noted inner setting issue is the leadership of the jail and the need for leaders to have an active role in endorsing, promoting, and providing resources (everything from staff to space to new protocols) to successfully administer medications to those in need.

Not unexpectedly, staff had varied opinions about MOUD, from supportive to the belief that these medications are merely substitutions of one drug with another. The interviews revealed that the stigma about MOUD was present in both correctional and medical staff had these varied attitudes, and that there was a general need to train both staff on the utility of the medications. Interagency collaborations were also often difficult, leading to several barriers because the medical and correctional staff seldom developed policies or procedures together; unresolved issues were encountered that needed attention. Some interagency collaborations mirrored the tensions with external treatment providers, and the requirement to offer medications in jail resulted in these tensions being aired about care coordination, procedures, and messages to the individuals in need of services (Matsumoto et al., 2022). The interviews revealed that more care coordination is needed to ensure that individuals get access to services after release including having jail staff more knowledgeable about community providers of medications. While peer navigators were advised, pretrial release individuals tend to underuse the navigators compared to convicted individuals on supervision; however, for some individuals, the navigators were an important part of continuing to use the medications in the community (Matsumoto et al., 2022).

## Juvenile Justice – Translational Research on Interventions for Adolescents in the Legal System (JJ-TRIALS) (see Knight et al., 2016; Belenko et al., 2022)

JJ-TRIALS was a multi-component, multisite suite of implementation interventions designed to improve the movement of youth through the Behavioral Health Services Cascade (Cascade) toward engagement and retention in evidence-based treatment (Belenko et al., 2017; Knight et al., 2016. The target client population was youth under community supervision (mainly probation). Staff from juvenile justice (JJ) and behavioral health (BH) partner agencies in 36 sites in 7 states. The intervention included a needs assessment and systems mapping exercise; staff training on behavioral health among justice-involved youth, treatment, interagency collaboration, data-driven decision-making procedures, goal selection support; and formation of local change teams/interagency workgroups to address agency goals around improving Cascade outcomes. A multisite cluster randomized design assigned 18 matched pairs of county-level community supervision agencies to receive either a Core set of implementation interventions or an Enhanced condition that included the Core intervention plus external facilitation of local change teams. Agency leadership in each study site selected a specific Cascade-related goal to address gaps in substance use treatment services for youth clients. JJ-TRIALS was organized around the **Exploration, Preparation, Implementation, Sustainment** (EPIS) framework (Aarons et al., 2011; Becan et al., 2018; Moullin et al., 2019), but JJ-TRIALS further elaborated the EPIS framework by considering a circular approach to implementation that can account for recursive movement through phases as needed (Becan et al., 2018).

JJ-TRIALS demonstrated that a complex implementation intervention could be successfully implemented over a multi-year period in a large number of sites. It was found that nearly half of youth were identified as being in need of treatment, but only about one-quarter of youth in need were referred to treatment (Belenko et al., 2022; Dennis et al., 2019; Wasserman et al., 2021). However, referrals increased among youth in need of treatment over baseline, and the Enhanced intervention yielded higher referrals over time (Belenko et al., 2022; see Table 3). However, although youth determined to be in need of treatment would ideally be referred to treatment as quickly as possible, results showed that less than half of referred youth received the referral within 30 days of initial screening (Wasserman et al., 2021).

**Table 3:**
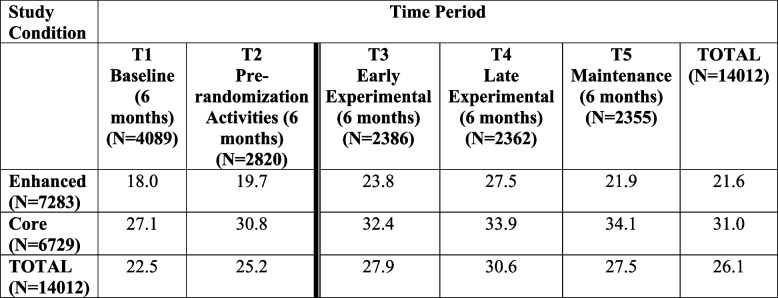
Percentage of youth with substance use treatment need who were referred to treatment, by time period and experimental condition

Dark line indicates randomization of sites into Core and Enhanced conditions

Chi-Square = 159.23, df = 1, *p* < .001

There was also an overall increase over time in initiation, engagement, and continuing care of treatment relative to baseline (Knight et al., 2022). Youth in the enhanced condition sites which received external facilitation of the local change team, initiated treatment earlier, and had greater penetration through the Cascade, compared with the Core intervention. Another key finding was that there was substantial variation in Cascade outcomes across sites, as well as variation in the relative impact of the Enhanced intervention (Belenko et al., 2022; DeLucca et al., 2022; Knight et al., 2022). These site differences highlight the importance of understanding the inner and outer context factors that can affect implementation and client outcomes.

The JJ-TRIALS intervention also resulted in reductions in youth recidivism. Recidivism among youth in the Enhanced condition sites decreased by 5.6 percentage points from baseline to the end of the experiment, compared with a two percentage point increase in the Core condition sites (Robertson et al., 2023). The interaction of experimental condition by time period was significant, indicating that the difference in recidivism between the experimental period and pre-experimental period was significant for the Enhanced sites: youth were 9% less likely to recidivate than Core condition youth in the experimental phases compared to pre-experiment. There were also significant differences in recidivism across study sites.

The JJ-TRIALS study illustrated how a conceptual implementation framework and multiple implementation interventions could be integrated into a complex system-level study design, agency goal selection, and outcomes reporting. The project demonstrated positive impacts of the implementation intervention on youth outcomes, and staff and agency acceptance of and participation in complex implementation strategies. The variations in outcomes across sites illustrated the complexity of implementation interventions and the importance of considering local inner and outer contexts in teasing out cross-site differences in both implementation and client outcomes.

## Discussion

IS focuses on examining the barriers and facilitators of innovations in organizational settings, as well as how these implementation-related issues affect a myriad of implementation, service, and client-level outcomes. IS frameworks such as CFIR and EPIS provide guidance for identifying these barriers and facilitators by focusing attention on the nature of the innovation, the inner and outer settings, the process of change, and the staff and organizations involved in the study. That is, the focus is on context from a wide-angle lens—looking into all aspects that affect what is implemented, how it is implemented, and where changes occurred that affect implementation. IS documents these barriers and facilitators using a myriad of methodologies, interventions, and measures, in a way that is not typical in process and outcome studies. The various implementation effectiveness designs specified by Curran et al. (2012) offer opportunities to test different phases of the process on various outcomes—the innovation, the change processes/implementation strategies, and the implementation outcomes.

The case studies highlighted in this article illustrate how IS can assist in identifying the implementation barriers and facilitators that account for variations in health outcomes for criminal legal system clients across different justice-health intercepts. While the working relationship among representatives from various organizations (often referred to as the change or policy team) is important, it is also clear from these studies that these are not easy relationships to navigate. Long-standing issues such as a difference in goals and agency missions affect implementation success, and the policy teams are a tool to address these differences and build better processes toward successful EBPT implementation (Belenko et al., 2022; Elkington et al., 2023; Molfenter et al., 2021). As noted by Mackey et al. (2024), working teams do not need to achieve consensus about a goal but need to continue to navigate operational issues to create seamless processes. Collaboration has long-term benefits in terms of identifying health system partners that are at least interested in the legal-involved population and see their role as ensuring that these individuals receive evidence-based care, who value serving the legal-involved populations, and who are committed to helping legal agencies have a defined role in service delivery. Teamwork is necessary regardless of whether there are legal mandates or resources dedicated to enhancing services. And, each of the different intercepts (from pre-arrest, arrest, pretrial, sentencing, jail, prison and community corrections) in the various settings has different cultural and historical issues that affects the ability to provide effective services to those that are involved in or likely to be involved in the justice system.

Legal mandates and additional resources are also two strategies that are important to both implementation and sustainability. Legal mandates illustrate stakeholder support for the innovation, as well as serving as a signal that the innovation has value. Even more so, such mandates often override personal opinions regarding the appropriateness of an innovation. Resources, of course, provide a visible indication that the innovation is valued. However, these resources need to be built on the infrastructure and not deplete existing resources (Taxman et al., 2025).

Identified barriers are often related to the perspective and opinions of the actors involved in the process as well as the constraining forces embedded in an innovation (such as cost, accessibility, or complexity). Legal actors are particularly sensitive to whether other agencies understand their emphasis on security, on dealing with breaches of security, the “dangers” of managing legal-involved populations, the needs of the courts and other agencies, legal constraints, and the limited resources that exist in legal settings. These perspectives often create tensions with other agencies. They also complicate efforts to create working relationships because legal organizations are often seeking empathy and special conditions given the environment in which they work. Of course, this is where leadership support for innovations from legal, health, and other agencies is critical to reinforce the importance of the working relationships and to address the problem at hand.

Work processes impact the ability of legal and health agencies to engage in “hand-off” procedures (e.g., transitioning individuals from one agency to another to provide care), especially across systems, and to provide interventions not directly related to security or the legal process such as medications for opioid use disorders or other substance use treatment. Typical barriers are the federal regulations associated with various medications (such as methadone or buprenorphine) which require modified procedures and resources for administering the medications—these place a burden on existing processes. Hand-off procedures are also complicated by the need to provide access to individuals, alter the work processes, and share protected health information about a client. While these organizational issues are not insurmountable, they do require both organizations to work differently—changes that are challenging to the existing environment.

The case studies also illustrated specific implementation strategies used to impact how the innovations are put in place. Examples include change teams, goal setting, service delivery pathways (using change teams), structured training and technical assistance, and various forms of collaboration. For the most part, the studies measured some of the changes, but only the Molfenter and colleagues (2021) study conducted an implementation-effectiveness trial comparing various implementation strategies. That study found that collaborations and working groups are hard to facilitate given the tensions among legal and non-legal organizations, past relationships, and priorities of various agencies. More information is needed to identify the efficacy of any given set of implementation strategies. In particular, the HEALing Communities studies were able to identify change strategies that make a difference (Chandler et al., 2023; Davis et al., 2024), such as public message campaigns, change teams, community organizers, champions, and so on. More attention is needed to document the change strategies and establish efficacy.

## A future research agenda

IS provides a new toolkit to advance our understanding of reforms that address innovations in various settings. In this paper, we focus on those innovations that were designed to improve client health-related service needs within the criminal legal system, particularly efforts to prioritize behavioral health services and treatments. The existing research establishes that IS can help facilitate a better appreciation for the context, culture, and climate of organizations involved in implementing innovations. However, this literature only scratches the surface of the research needed to maximize the impacts of IS. Based on a review of the literature, we would prioritize the following research priorities:

1. An emphasis on establishing the comparative efficacy of different implementation strategies on the perspectives and opinions of legal and/or health actors, collaboration among the agencies, fidelity to the innovation, and impact on client-level outcomes. For change strategies to be effective, there is a need to ensure that they address the host of implementation, service, and client-level outcomes both in terms of proximal and distal outcomes.
2. An emphasis on unraveling the “culture of control” (see Taxman, 2024) which fosters the tension between security and health-related innovations in legal settings. This culture is often a barrier to change due to the perspectives of legal/health actors, the existing regulations and procedures that interfere with providing services and treatments, and the perception that behavioral health (or medical services) are alien to a punishment system. Future studies should investigate how to address this culture, and then demonstrate impact on the individuals in terms of short-term impacts and long-term care.
3. Implementation requires the translation of science into the operational process but overall, we know little about how best to operationalize scientific knowledge into practice that is both acceptable and relevant to the opinions, perspectives, and beliefs of leaders, staff, and stakeholders.
4. The IS field has several frameworks that are useful to help on conceptualizing and measuring the change processes. The EPIS model is the most prevalent framework used. But little has been done to modify the EPIS or other models to accommodate the complex legal-health settings and issues. While CJ-IIM provides an emphasis on the multiple layers of stakeholders, this model has also not been fully tested. More attention is needed to identify change process frameworks that are most relevant and salient for the legal-health settings.
5. New innovations such as the automated clinical decision support systems tested in the eConnect project are emerging. Despite their promise, additional IS-focused research is needed to look more deeply into the impact of such systems on organizations, staff, service delivery, and client outcomes.
6. To date, most IS research in the criminal legal setting has focused on the implementation of EBPTs in correctional agencies, probation, and drug courts. However, many key decisions are made at the “front end” of the legal system that greatly affect health outcomes and access to services. The growing importance of police diversion and deflection policies, progressive prosecution and diversion, and specialty court programs are ripe for research on how these innovations can be implemented more effectively. In a recent paper, del Pozo et al. (2024) argued for the need to incorporate IS theories, measures, and interventions into policing research to further our understanding of implementation and de-implementation processes in that space.
7. Despite the growth of implementation research and growing support for funding such studies, little is known about the long-term sustainability of organizational innovation and change. Fidelity to and continued uptake of innovations tend to dissipate over time, especially after a research project has ended. Changes in agency leadership and staff turnover, or funding reductions often lead to innovation decay or de-implementation of an innovation. To achieve long-term cost-effectiveness and sustainment of positive client outcomes, it is crucial to understand how best to prepare organizations and systems to sustain innovations over time and insulate them from the inevitable changes in the inner and outer context factors that affect implementation success.
8. Expand the use of implementation research across the various intercepts where individuals who are involved in the justice system (or likely to be) can benefit from health-related services. IS can be a useful tool to identify the barriers and facilitators of quality services—which can be used to better understand how to address the prevention and/or treatment needs of individuals for the goals of reducing recidivism, improving health outcomes, and advancing an individual’s quality of life.

These are just a few of the potential future research agendas that can help pave a pathway for legal and/or health studies to advance service delivery. IS offers new tools, concepts, measures, and methods to move forward in efforts to improve the implementation of reform in legal and/or health settings and achieve desired improvements in both public health and public safety.

## Data Availability

No datasets were generated or analysed during the current study.
